# Analysis of changes in trends in the consumption rates of benzodiazepines and benzodiazepine-related drugs

**DOI:** 10.1186/s40545-017-0128-4

**Published:** 2018-01-16

**Authors:** Miguel Angel Fernández García, Antonio Olry de Labry Lima, Ingrid Ferrer Lopez, Clara Bermúdez-Tamayo

**Affiliations:** 1 Hospital Virgen de las Nieves, Av Doctor Oloriz 16, 18012 Granada, Spain; 2 Andalusian School of Public Health, Campus de la Cartuja s/n, Cuesta del Observatorio 4, 18010 Granada, Spain; 3 Ciber de Epidemiologia y salud Publica (CIBERESP), Madrid, Spain; 4 Instituto de Salud de Granada (Ibs-Granada), Granada, Spain; 5 Seville Primary Care Pharmacy Clinical Management Unit, calle Maria Galiana s/n, 41013 Seville, Spain

**Keywords:** Benzodiazepines, Primary health care, Therapeutics, Inappropriate prescribing

## Abstract

**Background:**

To analyse trends in the rates of consumption of benzodiazepine (BZD) anxiolytics, BZD hypnotics and non-BZD hypnotics and the association with contextual factors.

**Methods:**

Descriptive time series study. Units of analysis were monthly dose per inhabitant per day (DID) and dose per medical card per day(DCD) of benzodiazepine(BZD anxiolytics(BZD-A), BZD hypnotics(BZD-H) and non-BZD hypnotics(Non-BZD-H) between January 2006-December 2015. We analysed 6 primary healthcare districts(PHD) and used defined daily doses (DDDs) to calculate the monthly DIDs(overall and by ATC group). Trends and monthly percentage change (MPC) were analysed through joinpoint regression.

**Results:**

The annual DID increased by 26% overall, the trend was different across ATC groups. Consumption in BZD-A and BZD-H increased (27.1%,61.9%), consumption in Non-BZD-H decreased by 35%. There was high variability in DCD across the PHD, with an overall increase of 10.2%(5.7%-22.9%). By ATC, DCD increased by 10.4% in BZD-A(4.2%-22.2%) and by 44.2% in BZD-H(33.2%-76.5%). The overall DCD in the Non-BZD-H decreased by 42.1%(19.7%-50.8%). We found an initial upward trend in consumption of BZD-A until April/2008(monthly percentage change –MPC- +0.5%), followed by a slightly slower increase (+0.1%). No changes in trend were detected in BZD-H. In Non-BZD-H, we observed an upward trend until February/2013(+0.1%), followed by a sharp decrease until August/2013(−6.3%), and finally a slight decrease(−0.3%).

**Conclusions:**

BZD consumption has increased in the last decade, with variability across areas. The changes in trends do not coincide with the financial crisis, introduction of prescriptions by active ingredient, electronic prescriptions or copayment. The only decrease in the Non-BZD-H may be linked to an intervention.

## Background

Benzodiazepines (BZDs) are used to treat insomnia, anxiety and chronic back pain. BZDs have significant adverse effects, including memory loss, increased risk of accidents and falls, and dependency [1–3]. Although guidelines recommend psychological treatments as first-line therapy before pharmacological treatment [4], and that BZDs only be used in the short term (2-4 weeks) when symptoms are incapacitating [5–7], numerous studies have shown that BZDs are being over-prescribed for prolonged periods in many countries [8–10].

For several years, various initiatives have promoted the rational use of diagnostic and therapeutic resources to avoid unnecessary interventions and/or those with potential risks for patients in specific situations. These include the Choosing Wisely initiative (2009) [11] and the NICE ‘do not do’ recommendations (2011) [12]. In 2013, a new initiative, called the Commitment to Quality of Spanish Scientific Societies, was launched in Spain. As part of this programme, the Spanish Society of Family and Community Medicine (SEMFYC) published a series of recommendations that included “Do not prescribe benzodiazepines (or non-benzodiazepine hypnotics) for long-term use in patients with insomnia” [13].

BZDs were first used in clinical practice in the early 1960s [14], and since then have been one of the most-prescribed drug groups in most developed countries [15]. In Spain, BZD consumption has undergone sustained growth, from a defined daily dose (DDD) per 1000 inhabitants per day (DID) of 32.7 in 1992, to 56.7 in 2000, and 89.3 in 2012 [16, 17]. This 54.7% increase between 2000 and 2012 is beyond comparison with trends in any other countries in our setting with the exception of Portugal, where BZD consumption increased by 24% between 2003 and 2010 [17]. In all other countries, BZD consumption rates remained stable or even decreased, with a more marked reduction in anxiolytics than hypnotics [18–22].

According to the latest Annual Report of the Spanish National Health Service (2015), the 15 most-prescribed active ingredients include 3 benzodiazepines: 2 anxiolytics (lorazepam and alprazolam, 16.4 and 11.8 million bottles, respectively) and a sedative hypnotic (lormetazepam, 9.3 million bottles). Prescriptions for these 3 active ingredients alone had a retail value of 79.3 million euros during 2015 [23].

As evidence-based clinical guidelines [1] on BZD use are not being applied in clinical practice, we saw the need for an analysis of how prescribing trends have changed over recent years. The aim of this study is therefore to study trends in BZD consumption rates in the province of Seville and its primary healthcare districts (PHDs) and healthcare management areas (HMAs) between 2006 and 2015. We set out to quantify those trends, delimit the resulting trend periods and correlate changes to relevant events during the period, such as the start of the financial crisis, the introduction of prescriptions by active ingredient, electronic prescriptions and copayment, or safety alerts issued by regulatory agencies (FDA, AEMPS) [24, 25].

## Methods

### Study design

Descriptive time series study.

### Units of analysis

Total and disaggregated monthly dose per inhabitant per day (DID) and dose per medical card per day (DCD) of medicines derived from and related to benzodiazepine, in 3 therapeutic groups (BZD anxiolytics – N05BA (group BZD-A); BZD hypnotics – N05CD (group BZD-H); and non-BZD hypnotics – N05CF (group Non-BZD-H)) between January 2006 and December 2015.

### Setting

Four primary healthcare districts (Seville (DS), Aljarafe (DA), Seville North (DSN) and Seville South (DSS)) and 2 healthcare management areas (Osuna (HMAO) and Seville South (HMASS)) in the province of Seville, Spain. Those organizations serve 8.2% of the total population of the region of Andalusia and 1,5% of the whole of Spain. All inhabitants are covered by the Public Health System.

### Variables

#### Defined daily dose (DDD)

The average maintenance dose for a drug used for its main indication via one route of administration in adults [26]. The DDD is not the same as the recommended or prescribed dose. DDDs are established by the World Health Organization (WHO) and available on the website of the WHO Collaborating Centre for Drug Statistics*.*

#### Dose per card per day (DCD)

Defined as the DDD per 1000 medical cards per day. DCDs can be used to compare PHDs and HMAs with different population densities.

#### Dose per inhabitant per day (DID)

Defined as the DDD per 1000 inhabitants per day. The DID provides information about a population’s exposure to a particular drug or group of drugs. The annual DID can be calculated based on the number of packs/bottles dispensed using the following formula:

$$ \mathrm{DID}=\frac{\mathrm{U}\times \mathrm{PF}\times \mathrm{Q}\times 1000}{\mathrm{DDD}\times \mathrm{inhabitants}\times 365} $$

U = units (packs/bottles); PF = number of pharmaceutical forms per unit; Q = amount of active ingredient in each pharmaceutical form. The monthly DID can be calculated using the same formula, replacing the 365 days of the year with the number of days in the month studied. Data regarding prescriptions and treatment costs per day in the primary healthcare district of Seville were obtained from the Andalusian Public Health Service.

### Data source

DDDs and DCDs by ATC group were obtained based on data in the dispensing database of the Andalusian Health Service, which records all prescriptions dispensed by community pharmacies throughout Andalusia. We calculated DIDs using data from Seville’s provincial census, which gives the number of inhabitants per year on 1 January of that year [27].

The database includes the prescriptions detailed by age, sex, active drug principle, dose, dose schedule and duration of treatment. The quality of the database is acceptable, since the payment to community pharmacies depends in part on this.

### Data analysis

We used the DDDs to calculate the 120 monthly DIDs (overall and broken down by ATC group [28]) in the 10-year study period. We created graphs showing monthly trends and the differences between consumption rates in the first and last years, or in the first and last months of each period. We used joinpoint regression to explore trends using Joinpoint Trend Analysis Software. This statistical analysis program was developed by the Surveillance, Epidemiology, and End Results (SEER) Program of the National Cancer Institute to analyse trend joinpoints. We set the maximum number of joinpoints (changes in trend) for each analysis to 5.

## Results

### Consumption rates over time

**Fig. 1:**
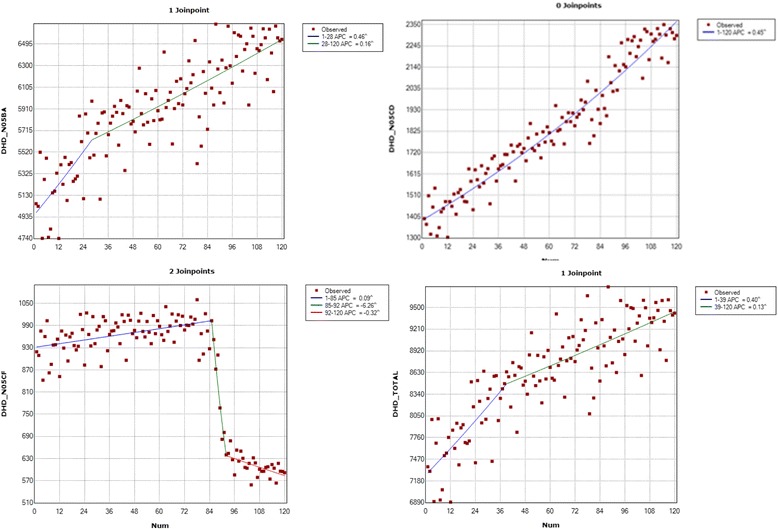
Seville provincial monthly DID (overall and by ATC group) 2006-2015. Axis x: Rates per 100,000 inhabitants. Axis y: months of the period

The annual DID increased by 26% overall. However, the trend was not equal across all ATC therapeutic groups: consumption rates of drugs in groups BZD-A and BZD-H increased by 27.1% and 61.9%, respectively, but consumption of drugs in the Non-BZD-H group decreased by 35%. The results were similar when we compared the variation in overall DID between January 2006 and 2015. There was an overall increase of 21.8% (29.3% in BZD- A, 64.5% in BZD-H). However, DID in the Non-BZD-H group decreased by 35.5% (Table 1 and Fig. 1).

**Table 1:** Seville provincial annual and monthly DID, differences and percentage increases, 2006-2015 (overall and by ATC group)

Annual DID	2006	2015	Difference: 2015-2006	Increase Difference/2006 (%)
N05BA	50.88	64.71	13.83	27.18
N05CD	14.09	22.82	8.73	61.95
N05CF	9.19	5.97	−3.22	−35.03
TOTAL	74.16	93.49	19.33	26.06
Monthly DID	Jan 2006	Dec 2015	Difference	Monthly DID
N05BA	50.55	65.37	14.82	29.31
N05CD	13.95	22.96	9.01	64.58
N05CF	9.19	5.92	−3.27	−35.58
TOTAL	73.70	94.25	20.55	21.80

N05BA, group BZD-A, benzodiazepine anxiolytics. N05CD, group BZD-H, benzodiazepine hypnotics. N05CF, group Non-BZD-H, non-benzodiazepine hypnotics

**Fig. 2:**
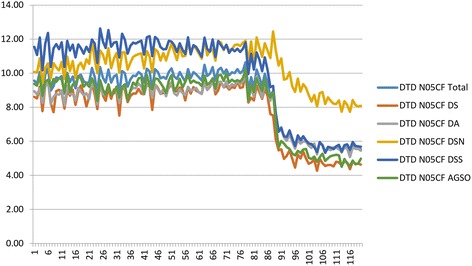
Monthly DCD of non-benzodiazepine hypnotics (N05CF), overall and by primary healthcare district/health management area, Seville, January 2006-May 2016

Between December 2015 and January 2006, there was high variability in DCD by therapeutic group across the different PHDs and HMAs. Overall DCD increased by 10.2%, but the increase ranged from 5.7% in DS to 22.9% in HMAO. By ATC group, overall DCD increased by 10.4% in group BZD-A (ranging from 4.2% in DS to 22.2% in HMAO), while in group BZD-H overall DCD increased by 44.2% (33.2% in DSN to 76.5% in HMAO). Finally, the overall DCD in the Non-BZD-H group decreased by 42.1% (19.7% in DSN to 50.8% in DSS) (Fig. 2).

### Joinpoint analysis

When we analysed the 120 monthly DIDs by therapeutic group, we found an upward trend in group BZD-A, with 2 distinct segments separated by a joinpoint. Between January 2006 and April 2008, the monthly percentage change (MPC) was 0.5%, while between April 2008 and December 2015 the MPC was slightly lower at 0.1%. No changes in trend were observed in the BZD-H group, where there was a sustained increase (0.4%) in DID. In the Non-BZD-H group we found 3 trend segments separated by 2 joinpoints. DID increased initially (MPC 0.1%) between January 2006 and February 2013. There was then a sharp decrease (−6.3%) between February 2013 and August 2013. Finally, there was a slight decrease (−0.3%) between August 2013 and December 2015 (Table 2).

**Table 2:** Changes in trends in monthly DID by ATC group, DCD by primary healthcare district/healthcare management area, and DCD of drugs in the Non-BZD-H group, province of Seville

PHD/HMA	Period (month/year)		MPC	Lower and upper limits		t	*p*
Monthly DID							
N05BA	01/06	04/08	0.5	0.3	0.7	4.5	< 0.0001
	04/08	12/15	0.1	0.1	0.2	7.0	< 0.0001
N05CD	01/06	12/15	0.4	0.4	0.5	40.7	< 0.0001
N05CF	01/06	02/13	0.1	0.1	0.1	4.6	< 0.0001
	02/13	08/13	−6.3	−8.1	−4.4	−6.4	< 0.0001
	08/13	12/15	−0.3	−0.5	−0.1	−3.2	< 0.0001
Total	01/06	03/09	0.4	0.3	0.5	6.8	< 0.0001
	03/09	12/15	0.1	0.1	0.2	7.0	< 0.0001
Monthly DCD							
D. Seville	01/06	06/11	0.2	0.2	0.3	7.8	< 0.0001
	06/11	12/15	−0.2	−0.3	−0.1	−5.5	< 0.0001
D. Aljarafe	01/06	03/12	0.2	0.2	0.3	11.3	< 0.0001
	03/12	12/15	−0.1	−0.2	−0.0	−2.8	< 0.0001
D. Seville North	01/06	09/11	0.3	0.3	0.4	13.3	< 0.0001
	09/11	12/15	−0.1	−0.2	−0.1	−3.6	< 0.0001
D. Seville South	01/06	09/11	0.3	0.2	0.3	11.3	< 0.0001
	09/11	05/16^b^	−0.2	−0.3	−0.2	−7.1	< 0.0001
HMA Osuna	01/06	11/11	0.3	0.3	0.4	14.0	< 0.0001
	11/11	12/15	−0.1	−0.2	−0.0	−2.6	< 0.0001
Total	01/06	09/11	0.3	0.2	0.3	10.4	< 0.0001
	09/11	05/16^b^	−0.2	−0.2	−0.1	−4.9	< 0.0001
Monthly DCDs of non-benzodiazepine hypnotics (group Non-BZD-H, N05CF)							
D. Seville	01/06	01/13	0.1	0.0	0.1	3.1	< 0.0001
	01/13	08/13	−8.4	−10.6	−6.1	−7.1	< 0.0001
	08/13	05/16	−0.2	−0.4	−0.0	−2.3	< 0.0001
D. Aljarafe	01/06	02/13	0.1	0.0	0.1	3.8	< 0.0001
	02/13	09/13	−6.2	−8.0	−4.4	−6.7	< 0.0001
	09/13	05/16	−0.3	−0.4	−0.1	−3.5	< 0.0001
D. Seville North	01/06	01/13	0.2	0.1	0.2	8.4	< 0.0001
	01/13	06/14	−1.9	−2.3	−1.4	−8.3	< 0.0001
	06/14	05/16	−0.2	−0.5	0.0	−1.7	0.1
D. Seville South	01/06	09/11	0.0	−0.0	0.1	0.9	0.4
	09/11	02/13	−0.6	−1.0	−0.2	−2.7	< 0.0001
	02/13	06/13	−10.7	−15.7	−5.3	−3.8	< 0.0001
	06/13	08/14	−1.2	−1.8	−0.6	−3.9	< 0.0001
	08/14	05/16	0.0	−0.3	0.3	0.0	1.0
HMA Osuna	01/06	03/13	0.0	−0.0	0.1	1.0	0.3
	03/13	06/13	−15.2	−24.8	−4.4	−2.7	< 0.0001
	06/13	10/15	−0.7	−1.0	−0.5	−7.1	< 0.0001
	10/15	05/16	2.0	0.4	3.6	2.4	< 0.0001
Total	01/06	01/13	0.1	0.0	0.1	3.1	< 0.0001
	01/13	08/13	−6.9	−8.7	−5.0	−7.0	< 0.0001
	08/13	05/16	−0.4	−0.5	−0.2	−4.7	< 0.0001
Monthly percentage change is significantly different from zero at alpha = 0.05							

PHD: Primary health District, HMA: healthcare management areas, MPC: monthly percentage change, DID: monthly dose per inhabitant per day, DCD: Dose per medical card per day, N05BA: group BZD-A, benzodiazepine anxiolytics: N05CD, group BZD-H: benzodiazepine hypnotics, N05CF: group Non-BZD-H: non-benzodiazepine hypnotics. Period: January 2006-May 2015

When we examined all groups together, the overall trend was upwards, with a single joinpoint in March 2009. There was an increase (MPC 0.4%) in the first segment, followed by moderate growth (0.1%) until December 2015. When we analysed the overall DCD in the different PHDs and HMAs, we found 2 segments divided by 1 joinpoint. In the first segment, DCD increased with an MPC of 0.2% (in DS and DA) or 0.3% (DSN, DSS and HMAO). In the second segment, DID decreased with an MPC of −0.1% (DA, DSN and HMAO) or −0.2% (DS and DSS), with the joinpoint between June 2011 (DS) and March 2012 (DA) (Table 2).

We observed an overall decrease in the Non-BZD-H group, and therefore carried out a detailed analysis of the monthly DCD trends in this therapeutic group by PHD/HMA. In the DS, DA and DSN districts we found 3 segments separated by 2 joinpoints. In the first segment, there was a slight upward trend with an MPC of 0.1% (DS and DA) or 0.2% (DSN) until January (DS and DSN) or February (DA) 2013. In the second segment, there was a decrease in DCD with an MPC of −1.9% (DSN), −6.2% (DA) and −8.4% (DS) until August 2013 (DS), September 2013 (DA) or June 2014 (DSN). There was a smaller decrease of −0.2% (DS and DSN) or −0.3% (DA) in the final segment. We should highlight that the first segment for DSS and HMAO is almost flat, i.e. the MPC is close to or equal to zero and not statistically significant. The first joinpoint for DSS is in September 2011, followed by a second, slight downward segment with an MPC of −0.6% until February 2013. The second joinpoint in DSS, in February 2013, marks the start of a sharp decrease in DCD (−10.7%). This increase lasts just 4 months, until June 2013. The decrease then becomes less extreme (−1.2%) until August 2014. The last segment, up to May 2016, is relatively flat and not statistically significant. In HMAO, after an initial flat segment, the second segment beginning in March 2013 shows a marked decrease (−15.2%), although it lasts for just 3 months. Its third segment starts in 2013 with a slight decrease (−0.7%) until October 2015. There is a fourth segment between October 2015 and May 2016, with an MPC of 0.2% (Table 2, Fig. 2).

## Discussion

This study shows that BZD consumption in the province of Seville has increased by more than 20% in 10 years. This finding is consistent with the results of a national study by the Spanish Ministry of Health, which found an annual increase of 2.5-3% between 2000 and 2011 with some spikes [17]. We conducted our study in the province of Seville, home to 23.1% of the population of the region of Andalusia [27]. This is a large sample, and our study is the first to break down consumption rates by primary healthcare district and healthcare management area, enabling a detailed analysis of trends. The increase detected in the rate of consumption of BZDs cannot be down solely to an ageing or growing population, as the increase in consumption is almost 5 times the increase in the number of inhabitants [27].

This study has some limitations which should be considered when interpreting the results. Firstly, we used 2 indicators for the analysis of trends: DID for provincial trends and DCD for the healthcare districts and healthcare management areas. Although they are very similar, DCD may have been underestimated as there may be users with medical cards who are not included in the census. When we analysed the increase by level of care, we found that the increase in primary care (overall DCD in PHDs/HMAs) was 10.2%, while the overall provincial increase (DID, primary and hospital care) was 21.8%. We can therefore assume that approximately 11.6% of the increase is attributable to hospital prescriptions. Because of the significant impact of such prescriptions (>50% of the increase), we recommend that studies of prescriptions in this level of care be conducted.

When we compare the overall DIDs for the province of Seville with those for Spain as a whole for the last available period (2006-12) [22], it appears that the growth has been slower (a provincial increase of 20.2% vs. a national increase of 26.4%), although the initial rates were higher (DID of 74.1 vs. 70.6). Both DIDs hit around 89 in 2012, and continued to increase in all subgroups. A study in Asturias had almost identical results [29]. Because we do not have the updated national data, we do not know if the Non-BZD-H group followed the same downward trend nationally as found in Seville from 2013 onwards. BDZs are one of the most commonly prescribed classes of psychotropic medications in developed countries [30] but rates of consumption in the last years remains high and stable. For example, in 2012, there were approximately 85 million BDZs prescriptions written in the United States to outpatients, which was not significantly changed from the 90 million written in 2001 In the Dutch population aged 55–64, overall b BDZs use remained stable from 1992 to 2012, with a high proportion of long-term users, despite the effort to reduce BDZs use and the renewal of the guidelines [19].

The relative importance of drugs in group BZD-A (>80% of prescriptions) means that the increase in the overall rate (26%) is almost exactly the same as the increase in that group alone (27.1%). This group cancels out the increase in the BZD-H group and decrease in the Non-BZD-H group. This can be seen in the trend graphs, where the trend for group BZD-A is very similar to the overall trend, although the latter is slightly modified by the decrease in Non-BZD-H consumption.

One noteworthy result is the significant decrease in consumption of drugs in the Non-BZD-H group. Zolpidem is the most-prescribed drug in this group, accounting for 90% of all prescriptions. In January 2013, the FDA [24] warned of the risk of using zolpidem at high doses. As a result, primary care pharmacists designed a training intervention for primary care doctors in the province of Seville. The aim was to raise awareness of the translated FDA alert, providing a list of patients affected and proposed interventions. Given the effectiveness of this initiative in the province of Seville [31], interventions of this sort should be extended to all healthcare professionals in both primary and hospital care settings, whose actions largely determine the success or failure of any healthcare intervention.

When we began our analysis, we expected the start of the global financial crisis (2009) [32] to cause an increase in the rates of consumption of these drugs. However, we observed no changes in trend consistent with this hypothesis. This result is consistent with those of studies in Paterna [33] and Asturias [29]. In fact, the only change observed was in March 2009, when the MPC of overall DID decreased from 0.4 to 0.1%. We also hypothesised that the introduction of copayment of medicines (July 2012) [34] would lead to a decrease in consumption. However, as in a previous study in Murcia [35], there were small decreases in the first few months but the rates bounced back quickly in the following months, with no overall change in trend. Electronic prescriptions and prescriptions by active ingredient were both introduced in 2005 and are now in widespread use. We expected the introduction of these measures to cause a change in BZD consumption. However, we observed no changes in trend consistent with this hypothesis.

The use of BZDs has increased in the last decade, with wide variability in the rates of BZD consumption within the province of Seville. The variability between neighbouring and homogenous small areas makes us hypothesize that the increase in use of BZDs is not only due to the increased prevalence of insomnia and due to other associated conditions.

The changes in trends do not coincide with the start of the financial crisis or the introduction of prescriptions by active ingredient, electronic prescriptions or co-payment. The only decrease in the Non-BZD-H group could be linked to a training intervention conducted with doctors, run in response to an alert issued by the US Food and Drug Administration (FDA) [25]. The multiple causes of the increase in BZD consumption need to be studied in detail, and further training interventions of this sort must be run.

## Conclusions

BZD consumption has increased in the last decade, with variability across geographical areas. The changes in trends do not coincide with the financial crisis, introduction of prescriptions by active ingredient, electronic prescriptions or copayment. The only decrease in the Non-BZD-H may be linked to an educational intervention for physicians.

## Abbreviations

BZD: Benzodiazepine
BZD-A: Benzodiazepine anxiolytics
BZD-H: Benzodiazepine hypnotics
HMAs: Healthcare management areas
Non-BZD-H: Non-benzodiazepine hypnotics
PHDs: Primary healthcare districts
